# Ruptured Left Ventricular False Tendon Mimicking a Mural Vegetation

**DOI:** 10.7759/cureus.11885

**Published:** 2020-12-03

**Authors:** Anuraag Sah, Sherif Elkattawy, Sahitya Posimreddy, Omar Elkattawy, Mirette G Habib

**Affiliations:** 1 Internal Medicine, Rutgers-New Jersey Medical Center/Trinitas, Elizabeth, USA; 2 Cardiology, Saint Joseph's University Medical Center, Paterson, USA; 3 Internal Medicine, New Jersey Medical School, Jersey City, USA; 4 Interventional Cardiology, Saint Michael’s Medical Center, Newark, USA

**Keywords:** vegetation, false tendon, thrombus

## Abstract

Left ventricular false tendons are cord like structures that traverse the left ventricular cavity. They are found in approximately half of the human hearts examined at autopsy and have no clinical or prognostic significance. They have been well described and usually pose no diagnostic dilemma. We present the first case of a partially ruptured false tendon mimicking mural vegetation in an 80-year-old male with extended-spectrum beta-lactamase Escherichia coli bacteremia.

## Introduction

Left ventricular false tendons (LVFTs) are distinct fibromuscular structures that are extensions of the innermost myocardial layer of the left ventricle and may vary in size and dimension [1]. They are generally benign anatomic variants that are remnants of the embryologic development of the four-chambered heart. Data regarding the prevalence, incidence, and clinical significance of LVFTs is largely from autopsy studies and two-dimensional echocardiographic studies from tertiary centers. The proposed incidence ranges from 18-26% in echocardiographic studies and 34% from autopsy studies [1]. Its clinical significance is described from a prospective Framingham study that determined that LVFTs were associated most commonly with innocent precordial murmurs and electrocardiographic (ECG) evidence of left ventricular hypertrophy, but no statistical significance or relevance was established with the risk of increased mortality secondary to cardiac etiology [‎1]. Although intact, LVFTs are readily identified on echocardiograms and have been established to serve no clinical significance, ruptured tendons in the cavity of the left ventricle may resemble vegetations, thrombus, or ruptured chordae tendineae and in an appropriate clinical setting may lead to false diagnosis and inappropriate management of a relatively benign finding [‎2]. Here, we describe an unusual case of a partially ruptured false tendon mimicking mural vegetation.

## Case presentation

We present a case of an 80-year-old male with a past history of hypertension, heart failure with reduced ejection fraction, type 2 diabetes mellitus, dyslipidemia, and acute coronary syndrome status post percutaneous coronary intervention with non-drug eluting stent placement in 2019. He presented with a three-day history of subjective fever associated with multiple episodes of non-bloody emesis and intermittent right-sided lower abdominal pain with radiation to the ipsilateral back. No additional complaints were elicited on the initial presentation. No flank tenderness was appreciated on physical examination.

The patient was febrile to 103.1° F with an elevated white blood count (WBC) of 14,600 per microliter. He was hemodynamically stable and started on empiric broad-spectrum antibiotics. CT abdomen was significant for mild perinephric stranding but negative for calculi. Urine and blood cultures on admission were positive for extended spectrum beta-lactamases (ESBL) Escherichia coli and he was transitioned to meropenem given the sensitivity profile. 

**Figure 1:**
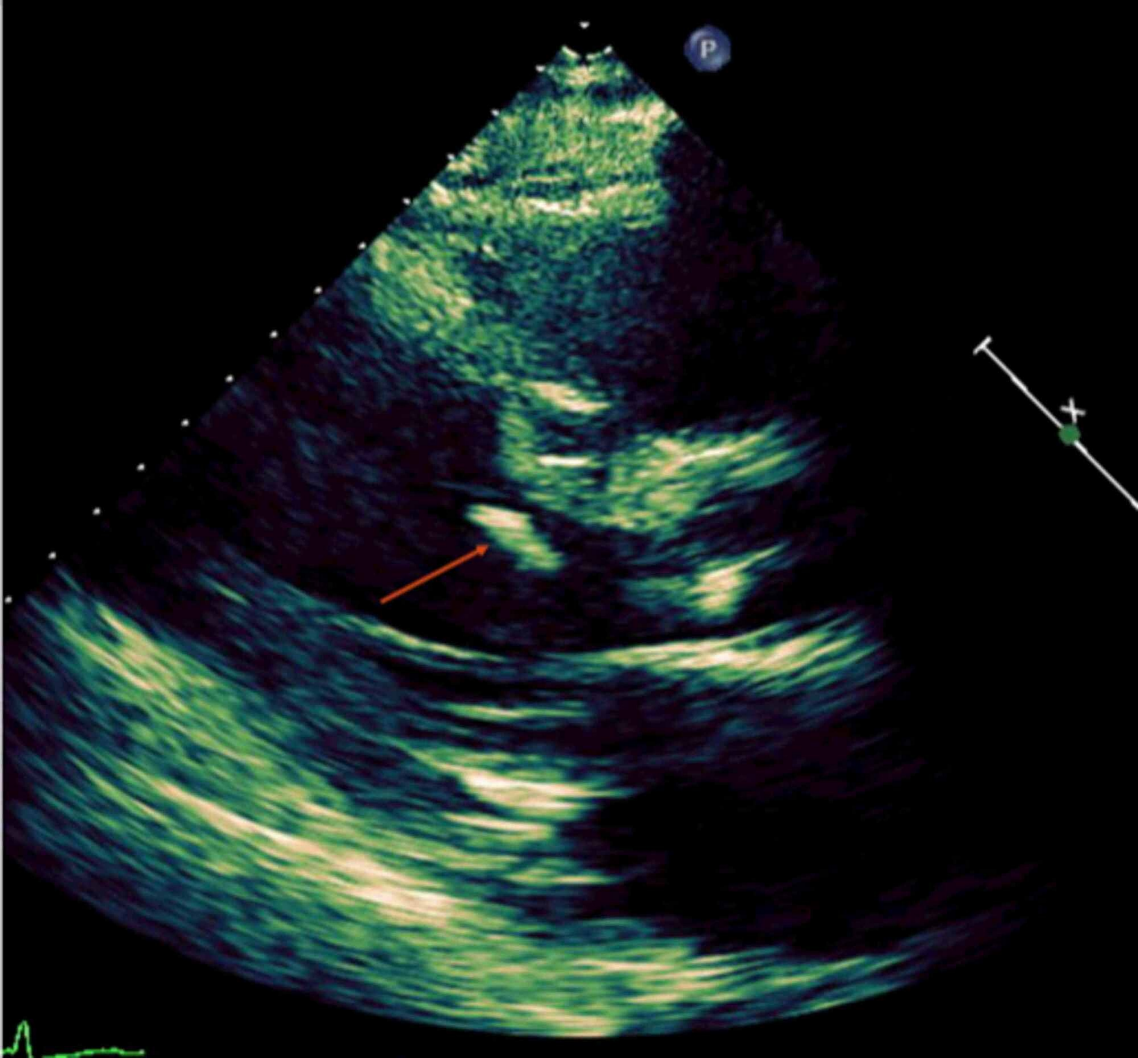
Transthoracic echocardiogram showing mobile hyperechoic echodensity (red arrow) near the basal septum of the left ventricle

The hospital course was complicated by a transient episode of dyspnea, which responded to diuretics. An electrocardiogram done at that time showed sinus rhythm with non-specific ST-T-wave abnormalities unchanged from prior. Transthoracic echocardiogram (TTE) was done because of the change in clinical status that showed an ejection fraction of 25-30% with severely decreased global left ventricular systolic function with aneurysmal anterior and anteroseptal walls unchanged from prior TTE. However, there was a new finding of a mobile echodensity (Figure 1) attached to the basal septum of the left ventricle.

**Figure 2:**
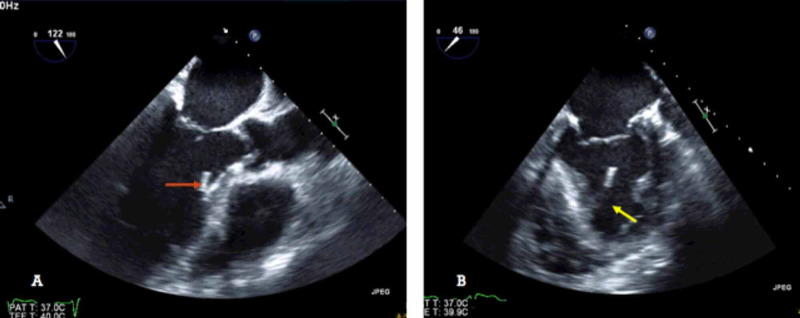
Transesophageal echocardiogram A: showing a smooth, sharp, linear echodensity (red arrow) not consistent with vegetation. B: an off-axis image showing the echodensity, part of the false tendon (yellow arrow).

Given the history of ESBL bacteremia, a transesophageal echocardiogram (TEE) was performed to further characterize the echodensity and to rule out infective endocarditis. It showed a linear mobile hyperechoic opacity with sharp borders, not characteristic of endocarditis (Figure 2).

**Figure 3:**
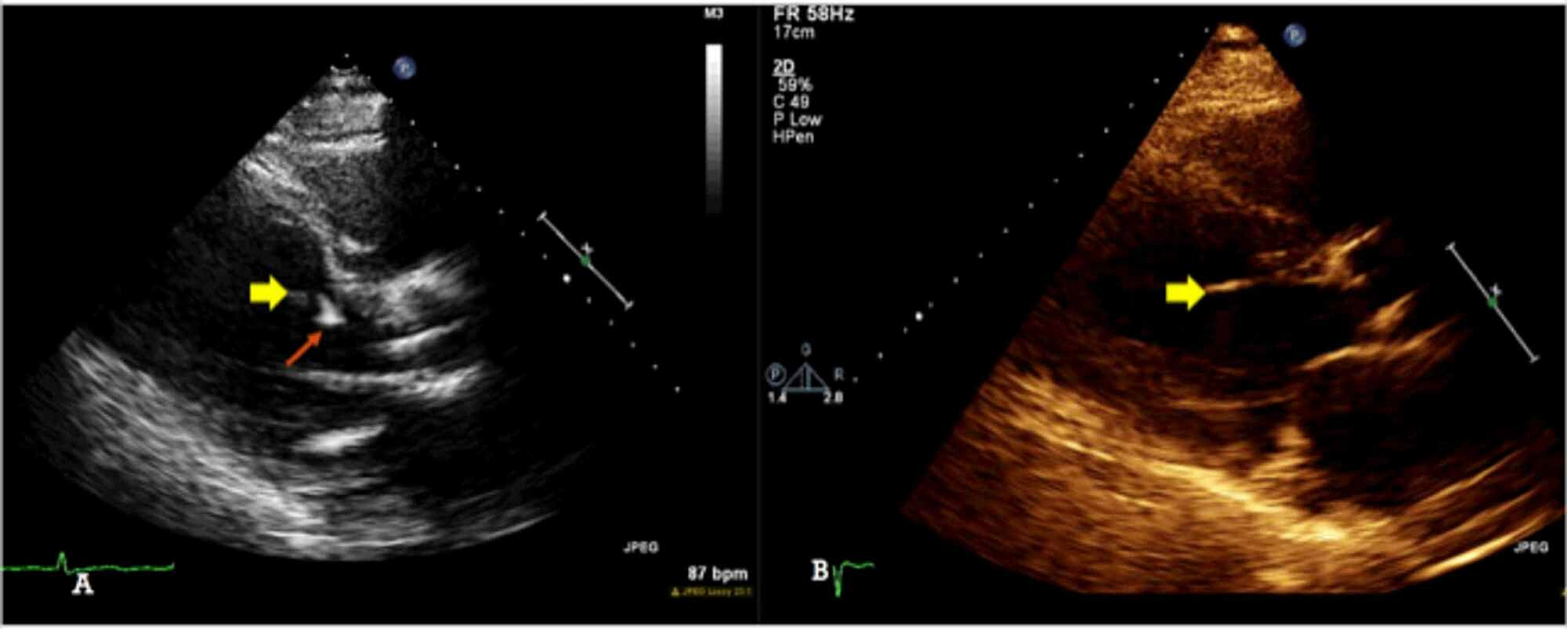
Comparison between current and prior transthoracic echocardiogram A: current transthoracic echocardiogram shows a linear structure (yellow arrow) next to the echodensity (red arrow). B: prior echocardiogram from one year ago shows a hyperechoic false tendon in the same location as the linear structure from the current echocardiogram.

The location of the linear density, when compared to prior echocardiograms, was consistent with a partially ruptured false tendon (Figure 3 current and prior TTE). Repeat blood cultures on appropriate antibiotic therapy were negative. 

## Discussion

Although LVFTs have been a well-established echocardiographic finding widely reported in the literature, in almost all reported cases, they remain attached as they traverse the interventricular septum and left ventricular free wall or papillary muscles without any connection to mitral valve leaflets [2]. In only one case report, LVFT was found to be dissociated from the ventricular wall; however, the rupture of the LVFT was secondary to endocarditis due to brucellosis infection [3]. There have been no case reports describing a partially ruptured LVFT.

As mentioned earlier, even though LVFTs are a benign finding and are readily seen on echocardiograms, partially ruptured LVFTs may resemble vegetation or thrombus, resulting in unnecessary therapies including anticoagulation or extended antibiotics. It is hard to distinguish between a partially ruptured LVFT and vegetation on a transthoracic echocardiogram; our case demonstrates the importance of comparing current studies with prior studies, which provide additional clues and help make a more informed diagnosis. 

It is also important to use additional tests in cases where a clear diagnosis cannot be made on TTE. A TEE has increased sensitivity compared to TTE and can help characterize the opacities better. Adequate description and characterization of echodensity on echocardiogram with commentary on tissue texture and attachment site may help differentiate between different structures [3].

## Conclusions

LVFT are cord-like structures that cross the left ventricular cavity and serve no prognostic significance. Our case report illustrates a very rare entity of an idiopathic primary partial rupture of LVFT and the importance of a systematic approach in coming to a correct diagnosis. We urge other researchers to pay close attention to the aforementioned finding, given its subtlety. 
